# Length and Redundancy of Outpatient Progress Notes Across a Decade at an Academic Medical Center

**DOI:** 10.1001/jamanetworkopen.2021.15334

**Published:** 2021-07-19

**Authors:** Adam Rule, Steven Bedrick, Michael F. Chiang, Michelle R. Hribar

**Affiliations:** 1 Department of Medical Informatics and Clinical Epidemiology, Oregon Health & Science University, Portland; 2 National Eye Institute, National Institutes of Health, Bethesda, Maryland

## Abstract

**Importance:**

There is widespread concern that clinical notes have grown longer and less informative over the past decade. Addressing these concerns requires a better understanding of the magnitude, scope, and potential causes of increased note length and redundancy.

**Objective:**

To measure changes between 2009 and 2018 in the length and redundancy of outpatient progress notes across multiple medical specialties and investigate how these measures associate with author experience and method of note entry.

**Design, Setting, and Participants:**

This cross-sectional study was conducted at Oregon Health & Science University, a large academic medical center. Participants included clinicians and staff who wrote outpatient progress notes between 2009 and 2018 for a random sample of 200 000 patients. Statistical analysis was performed from March to August 2020.

**Exposures:**

Use of a comprehensive electronic health record to document patient care.

**Main Outcomes and Measures:**

Note length, note redundancy (ie, the proportion of text identical to the patient’s last note), and percentage of templated, copied, or directly typed note text.

**Results:**

A total of 2 704 800 notes written by 6228 primary authors across 46 specialties were included in this study. Median note length increased 60.1% (99% CI, 46.7%-75.2%) from a median of 401 words (interquartile range [IQR], 225-660 words) in 2009 to 642 words (IQR, 399-1007 words) in 2018. Median note redundancy increased 10.9 percentage points (99% CI, 7.5-14.3 percentage points) from 47.9% in 2009 to 58.8% in 2018. Notes written in 2018 had a mean value of just 29.4% (99% CI, 28.2%-30.7%) directly typed text with the remaining 70.6% of text being templated or copied. Mixed-effect linear models found that notes with higher proportions of templated or copied text were significantly longer and more redundant (eg, in the 2-year model, each 1% increase in the proportion of copied or templated note text was associated with 1.5% [95% CI, 1.5%-1.5%] and 1.6% [95% CI, 1.6%-1.6%] increases in note length, respectively). Residents and fellows also wrote significantly (26.3% [95% CI, 25.8%-26.7%]) longer notes than more senior authors, as did more recent hires (1.8% for each year later [95% CI, 1.3%-2.4%]).

**Conclusions and Relevance:**

In this study, outpatient progress notes grew longer and more redundant over time, potentially limiting their use in patient care. Interventions aimed at reducing outpatient progress note length and redundancy may need to simultaneously address multiple factors such as note template design and training for both new and established clinicians.

## Key Points

**Question:**

How did the length and redundancy of outpatient progress notes change from 2009 to 2018?

**Findings:**

In this cross-sectional study of nearly 3 million outpatient progress notes written across 46 specialties at an academic medical center, median note length increased 60.1% from 401 words in 2009 to 642 words in 2018 while median note redundancy (ie, the proportion of text identical to the patient’s last note) increased 10.9 percentage points from 47.9% in 2009 to 58.8% in 2018. Both increases were significant.

**Meaning:**

In this study, outpatient progress notes grew longer and more redundant between 2009 and 2018, potentially limiting their use in patient care.

## Teaser

This cross-sectional study examines whether author experience and method of note entry are associated with the length and redundancy of progress notes among multiple medical specialties.

## Introduction

In 2009, the Health Information Technology for Economic and Clinical Health (HITECH) Act incentivized electronic health record (EHR) adoption in the US at a massive scale.^1^ The ensuing migration from paper to digital records transformed US health care, improving medication safety and guideline adherence while providing a wealth of data for quality improvement and research.^2,3,4,5,6^ However, EHR adoption has also been accompanied by sweeping regulatory change and growing concern that clinicians spend too much time documenting. Many clinicians spend more time interacting with EHRs than with patients.^7,8,9^ Much of that time is spent reading and writing clinical notes, which clinicians claim have grown longer, less informative, and less useful for patient care over time.^10,11,12,13^

Note bloat—the lengthening of notes with repetitive or clinically unhelpful text—has been widely discussed,^10,11,12,13,14,15,16^ but there is limited evidence on its scope or causes.^17,18^ Epic Systems estimated their clients’ notes doubled in length between 2009 to 2018,^18^ but no prior work has transparently quantified note bloat at scale. Evidence on causes is similarly lacking, although commentaries have pinned note bloat on exacting documentation requirements, such as the Centers for Medicare & Medicaid Services’ (CMS) Evaluation and Management codes, and indiscriminate copying of note text.^10,11,12,13,18,19,20,21^ Perceived consequences include notes taking longer to write, being more difficult to review, and being less accurate sources of data for quality improvement and research,^13^ although these outcomes are difficult to measure quantitatively.

A lack of evidence on note bloat’s scope, causes, and effects has not stopped organizations from responding. Recent position papers from the American College of Physicians,^14^ American Medical Informatics Association,^15^ and Association of Medical Directors of Information Systems^16^ all called for policy changes to address note bloat. CMS subsequently reduced their documentation requirements for outpatient visits starting in 2019, with additional changes taking effect in 2021.^22,23^ Some organizations have also set policies limiting note copying.^24,25^

It is hoped these changes will reduce both note bloat and documentation time. Yet, while there is growing evidence on typical documentation times,^7,8,9^ there is not a similar baseline for note lengths or how they have changed over time to compare the impact of recent policy changes against. Nor is there clear evidence that billing and copy-pasting are the only factors driving note bloat. This study begins to address these knowledge gaps by examining the length and redundancy of nearly 3 million outpatient progress notes written at an academic medical center between 2009 and 2018—the decade after the passage of the HITECH Act—and how these measures of note bloat associate with encounter attributes, clinician characteristics, and method of note entry.

## Methods

### Setting

This study was conducted at Oregon Health & Science University (OHSU), a large academic medical center in Portland, Oregon. OHSU implemented an institutionwide EHR in 2005 to 2006 (EpicCare; Epic Systems) and has used the same EHR since. This study adhered to the Declaration of Helsinki^26^ and was approved by the OHSU institutional review board, which granted a waiver of informed consent for analysis of EHR data. We followed the Strengthening the Reporting of Observational Studies in Epidemiology (STROBE) reporting guideline.

### Data Collection

This study’s primary hypothesis was that note length and note redundancy increased from 2009 to 2018. Secondary hypotheses included that most note text was templated or copied by 2018 and that higher rates of templating and copying associate with longer and more redundant notes. To test these hypotheses, we leveraged an existing corpus of 200 000 randomly selected patient records covering the decade from January 1, 2009, to December 31, 2018.^27^ This corpus was created by randomly sampling the records of patients with 2 or more primary care visits at OHSU before January 1, 2019. From this corpus, we collected the progress note, billing level, and new patient status for every outpatient office visit in 46 specialties. We excluded nonoffice visits from analysis, such as telephone encounters. Specialties with fewer than 1000 outpatient progress notes in the corpus were also excluded from analysis. To measure author experience, we queried OHSU’s data warehouse to identify when each author started using OHSU’s EHR—a proxy for when they joined OHSU—and whether they were a resident or fellow at the time of the visit. Data were not collected on authors' race or ethnicity as these were not consistently available. To measure the source of note text we collected metadata, only available for 2017 to 2018, tracking the number of characters in each note that were directly typed, copied, or templated and the number entered by the note’s primary author vs another member of the care team (eg, a technician or scribe).

### Calculating Note Length, Redundancy, and Text Source

The primary study measures were note length, note redundancy, and the text source. Because note bloat refers to both increasing length and decreasing informativeness, we measured both note length and redundancy; that is, how much of a note’s text occurred in prior notes and thus potentially provided little new information. We defined note length as the number of words in a note. Any continuous string of characters containing at least one letter or number was considered a word. We defined note redundancy as the proportion of a note’s words that occurred in the exact same order in the patient’s last note written by the same author. As in prior work, we used sequence alignment to identify identical sequences of text across notes.^28^ We only computed redundancy for notes where the patient’s last visit in a particular specialty had been with the same clinician and documented by the same author, providing a similarly constructed note for comparison. We did not compute redundancy for notes where the patient had no prior outpatient encounter in that particular specialty, or the most recent note had been written by another author. For notes written in 2017 to 2018, we determined text source by using EHR metadata to measure the proportion of note text that had been typed, templated, or copied. For each visit we also counted the number of prior outpatient visits documented in the EHR for that patient so we could model the impact of accruing EHR data on note redundancy and length.

### Statistical Analysis

We reported median values for note length and redundancy, which were nonnormally distributed. We reported mean values for the sources of note text to ensure the proportions of typed, templated, and copied text summed to 100%. We used cluster bootstrapping, which accounted for correlations between notes written by the same author or in the same specialty, to generate CIs for these measures in individual years.^29^ We also measured the change in median note length and redundancy between 2009 and 2018. When generating CIs for these changes over the decade, we used stratified 2-sample cluster bootstrapping to account for correlation between notes written by the same author in different years.^30^ This approach ensured each bootstrapped sample contained the same number of authors as the original sample in each specialty who wrote notes only in 2009, only in 2018, or both 2009 and 2018, thus mimicking observed personnel changes. CIs were generated across all specialties and 4 mutually exclusive specialty subgroups of primary, surgical, pediatric, and adult specialty care. Specialties were categorized based on the Association of American Medical College’s specialty descriptions (see eTable 1 in the Supplement).^31^ Bootstrapped CIs were set at 99% rather than 95% to account for subgroup analysis.

We developed 2 sets of multivariable mixed-effects linear models to examine factors associated with longer and more redundant notes. The first set modeled note length and redundancy across 2009 to 2018 as a function of year, visit billing level, visit (new or return patient status), author start year, author trainee status, and the patient’s number of prior outpatient visits. These decade-long models included random intercepts and random encounter year slopes for each author and specialty to account for correlations between notes written by the same author or in the same specialty. Because metadata on text source were only available for 2017 to 2018, we developed a second set of 2-year models that were identical to the decade-long models but only included notes written in 2017 to 2018 and added the percentage of copied, templated, and nonprimary-author text as fixed effects.

In both models of note length, we modeled the logarithm of note length to produce parameter estimates in terms of the percentage of note length gained or lost, rather than the absolute number of words gained or lost, making changes more comparable between specialties with different note lengths. Because note redundancy is a percentage, we reported redundancy gains or losses in percentage points. All models were built in R statistical software version 3.4.3 (R Project for Statistical Computing) using the lme4 library (version 1.1.15). Model estimates for fixed effects were reported with 95% Wald CIs. CIs that did not overlap with 0 were considered to show significant changes in note length or redundancy, or significant associations between model factors and note length or redundancy. Statistical analysis was performed from March to August 2020.

## Results

### Note Length

**Figure 1.**
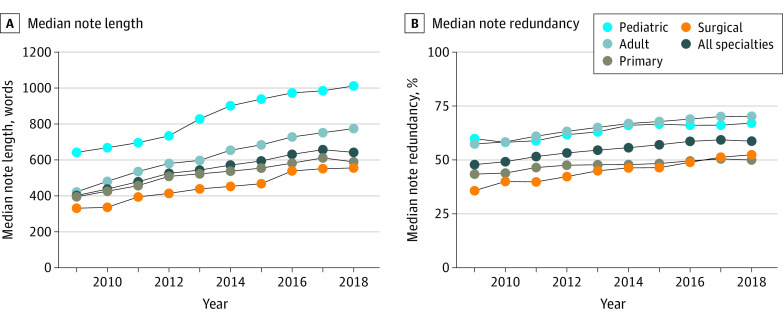
Median Note Length and Median Note Redundancy for All Specialties and by Specialty Type, 2009-2018

A total of 2 704 800 notes written by 6228 authors across 46 specialties were analyzed for length, representing nearly one-third (32.9%) of all outpatient office visits conducted at OHSU between 2009 and 2018. See eTable 1 in the Supplement for sample size by specialty. Median note length increased 60.1% (99% CI, 46.7%-75.2%) across all specialties from a median of 401 words (IQR, 225-660 words) in 2009 to 642 words (IQR, 399-1007 words) in 2018 (Figure 1A). Median note length increased significantly in all 4 subgroups, increasing 49.5% in primary care (99% CI, 33.3%-65.8%), 84.1% in adult specialties (99% CI, 55.9%-121.6%), 57.8% in pediatric specialties (99% CI, 33.9%-80.5%), and 68.2% in surgical specialties (99% CI, 35.8%-109.5%). See eTable 2 in the Supplement for note length by specialty.

### Note Redundancy

A total of 1 052 445 notes were analyzed for redundancy. Median note redundancy increased 10.9 percentage points across all specialties between 2009 and 2018 (99% CI, 7.5-14.3 percentage points) from 47.9% (IQR, 25.1%-67.0%) in 2009 to 58.8% (IQR, 37.7%-73.6%) in 2018 (Figure 1B). Median note redundancy increased significantly in each subgroup, increasing 6.5 percentage points (99% CI, 2.2-11.6 percentage points) in primary care, 12.8 percentage points (99% CI, 6.8-17.0 percentage points) in adult specialties, 7.2 percentage points (99% CI, 0.2-13.3 percentage points) in pediatric specialties, and 16.6 percentage points (99% CI, 9.2-23.6 percentage points) in surgical specialties. See eTable 2 in the Supplement for note redundancy by specialty.

### Text Source

**Figure 2.**
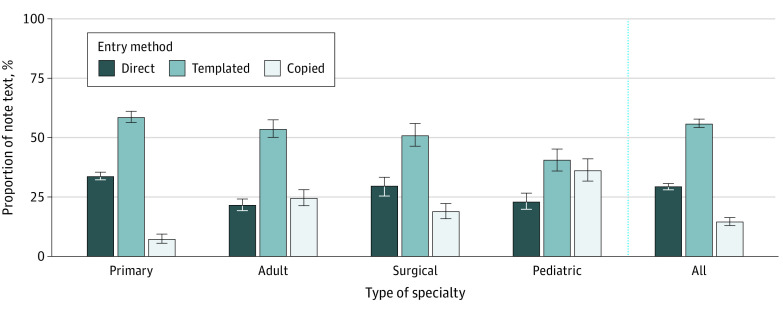
Percentage of Directly Typed, Templated, and Copied Note Text in 2018 by Specialty Type Error bars denote bootstrapped 99% CIs.

A total of 644 917 notes written in 2017 to 2018 were analyzed for text source. Across all specialties, a mean of 29.4% (99% CI, 28.2%-30.7%) of note text was directly typed in 2018 with 14.7% (99% CI, 13.1%-16.5%) copied and 55.9% (99% CI, 54.1%-57.7%) templated (Figure 2). In each subgroup, less than half of note text was directly typed in 2018, comprising a mean of 33.8% (99% CI, 32.1%-35.6%) of text in the average primary care note, 21.7% (99% CI, 19.5%-24.0%) in adult specialties, 23.1% (99% CI, 19.7%-27.5%) in pediatric specialties, and 29.8% (99% CI, 26.2%-34.2%) in surgical specialties. See eTable 2 in the Supplement for note text source by specialty.

### Models of Note Length and Redundancy

**Figure 3.**
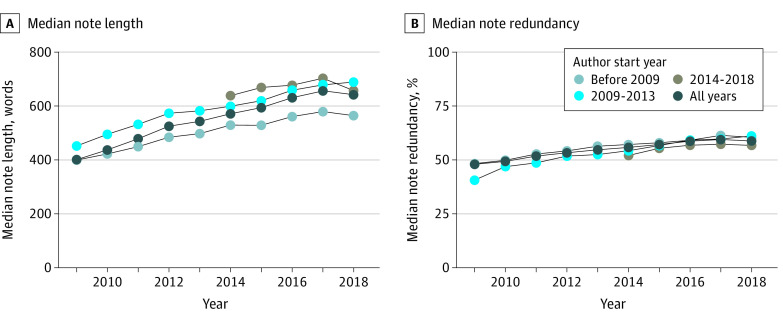
Median Note Length and Median Note Redundancy, Stratified by the Year Each Clinician Started Using the Electronic Health Record, 2009-2018

Modeled across 2009 to 2018 and controlling for other model factors, note length increased 2.4% (95% CI, 1.4%-3.5%) each year and 1.8% (95% CI, 1.3%-2.4%) for each additional year after 2005 that the note’s author started working at OHSU (ie, first used OHSU’s EHR) (Table 1). This combination of time and cohort factors is shown in Figure 3A. Residents and fellows were also modeled as writing 26.3% (95% CI, 25.8%-26.7%) longer notes than nontrainees. The 2-year model further found that each 1% increase in the proportion of copied or templated note text was associated with 1.5% (95% CI, 1.5%-1.5%) and 1.6% (95% CI, 1.6%-1.6%) increases in note length, respectively (Table 2). See eTable 3 in the Supplement for all model parameter estimates.

**Table 1.** Model Parameters for the Decade-Long Models of Note Length and Redundancy, 2009-2018

Factor	Note length increase, % (95% CI)	Note redundancy increase (95% CI), percentage point
Encounter year (per year)	2.4 (1.4 to 3.5)	0.7 (0.5 to 1.0)
Author start year (per year)	1.8 (1.3 to 2.4)	0.2 (0.03 to 0.3)
Author is resident or fellow	26.3 (25.8 to 26.7)	−7.0 (−6.7 to −7.3)
Billing: level 4 vs level 3	27.9 (27.7 to 28.2)	−1.9 (−1.8 to −2.0)
Billing: new vs return patient^a^	32.2 (31.9 to 32.4)	−16.7 (−16.2 to −17.1)
No. of prior visits (per visit)	0.1 (0.1 to 0.1)	0.04 (0.04 to 0.04)

^a^ Return visits where patients last saw their clinician 3 or more years previously were still billed as new patient visits, enabling analysis of the redundancy of 7369 notes written for return new patient visits.

**Table 2.** Model Parameters for 2-Year Models of Note Length and Redundancy, 2017-2018^a^

Factor	Note length increase, % (95% CI)	Note redundancy increase (95% CI), percentage point
Proportion of note text by nonprimary author (per 1%)^a^	0.5 (0.5 to 0.5)	−0.03 (−0.03 to −0.04)
Proportion of note text copied (per 1%)	1.5 (1.5 to 1.5)	0.6 (0.6 to 0.6)
Proportion of note text templated (per 1%)	1.6 (1.6 to 1.6)	0.4 (0.4 to 0.4)

^a^ See eTable 3 in the Supplement for all model parameters.

Modeled across 2009 to 2018 and controlling for other model factors, note redundancy increased 0.7 percentage points (95% CI, 0.5-1.0 percentage points) each year and 0.2 percentage points (95% CI, 0.0-0.3 percentage points) for each additional year after 2005 that the note’s author started working at OHSU. Notes written by trainees were 7 percentage points (95% CI, 6.7-7.3 percentage points) less redundant than those written by nontrainees (Table 1). The 2-year model found each 1% increase in the proportion of copied and templated note text was associated with increases in redundancy of 0.6 percentage points (95% CI, 0.6-0.6 percentage points) and 0.4 percentage points (95% CI, 0.4-0.4 percentage points), respectively (Table 2).

## Discussion

This study presents a large-scale cross-specialty analysis of note bloat. Its primary finding is that outpatient progress notes grew significantly longer and more redundant between 2009 and 2018, increasing 60% in length and 11 percentage points in redundancy over the decade. Although this is less than the doubling in length reported by Epic Systems over the same period,^18^ methods were not presented for that estimate, making the figures difficult to compare. Also, whereas previous single-year, single-specialty studies found outpatient pharmaceutical notes written in 2009 and outpatient ophthalmology notes written in 2018 both averaged 74% redundancy,^32,33^ this study measured redundancy across specialties and time. Notably, in 38 of the 46 observed specialties median note redundancy exceeded 50% in 2018, meaning more of the note’s text was identical to the patient’s last note than was novel. Longer and more repetitive notes are not harmful per se, but to the extent in which they support or hinder the varied uses of clinical notes. In this light, the dramatic observed increases in note length and redundancy are alarming if, as many clinicians and medical associations have argued, the primary purpose of notes is to support communication and patient care by concisely summarizing the patient’s condition, the care team’s actions, and the author’s thoughts.^11,12,13,14,15,16,19^ Long or repetitive notes can obscure critical patient information, hindering narrative and contributing to diagnostic errors.^34^ Bloated notes can also have impacts beyond direct patient care, such as taking longer to write or being a poor source of data for quality improvement and research.^13,35^

The second key finding is that a large portion of note text was generated using templates and copy-paste. In all specialties, less than half of note text was directly typed in 2018; and in 36 specialties, it was less than one-third. In this respect, this study extends 2 prior single-specialty studies, 1 in inpatient internal medicine and 1 in outpatient ophthalmology, which respectively found that only 18% and 9% of note text was directly typed.^36,37^ Numerous studies have highlighted how copying note text can propagate outdated information and contribute to diagnostic errors.^21,24,25,34^ However, this study observed nearly 4 times as much text written in 2018 was templated as was copied (55.9% vs 14.7%). Templates can reduce documentation time and increase standardization, but can also add potentially irrelevant information or introduce errors, as when used to insert default examination findings which were not actually observed.^38,39^ More attention should be paid to how clinicians use note templates, how they are governed, and their impact on patient care.^40,41,42^

The third key finding was the unexpected finding that note length was associated with multiple measures of author experience. As hypothesized, increased templating and copying of note text was associated with longer and more redundant notes. However, residents and fellows also wrote notes that were 26.3% longer than nontrainees, and each year later an author joined OHSU (ie, started using OHSU’s EHR) was associated with a 1.8% increase in note length. Compounded over time, clinicians who joined OHSU in 2018 would be expected to write notes that were 26% longer than those written in the same year by a similar clinician who joined in 2005. The causes of this cohort association are unclear. Future work might explore whether this cohort association extends beyond note lengths to differences in note contents (eg, inclusion of imported medication lists, problem lists, or laboratory results).

The simultaneous significant association of note length with time, cohort, trainee status, and method of text entry suggests the factors driving note bloat are multiple, interwoven, and complex. Policies and practices aimed at reducing note bloat will likely need to be similarly multifaceted. Prior studies and commentaries have floated potential interventions ranging from redesigning note templates to changing billing policies.^13,14,15,16,18,19,24,25,43,44^ Of the proposed interventions, redesigning note templates has the strongest supporting evidence. One multicenter study found that introducing a standardized note template and educating residents about documentation best practices led internal medicine residents to write shorter, more timely, and higher quality notes.^44^ Two single-center studies of standardized pediatric note templates found similar results.^43,45^ These studies recommend minimizing automatic importing of large data fields like medication lists and designing text prompts to encourage “novel independent input.”^13,43,44^

### Limitations

This study has several limitations that future work could address. First, it was an observational study of a sample of patient records. This sample covered about one-third of all outpatient office visits conducted at OHSU during the study period, but the observational design nonetheless precludes claims about causality. Second, this study looked at outpatient progress notes at a single academic medical center using one EHR, so it may not generalize to other note types, settings, or EHR systems. Third, data on the source of note text were only available for 2017 to 2018, limiting our ability to observe changes in this factor over time. Fourth, this study did not link note bloat to clinical outcomes, which would require an experimental design and additional data collection. Fifth, there are other factors that may associate with note bloat, such as practice location, physician training, documentation policies, or template design, which this work did not assess. Broadly, future work might explore how user interface, note template, and policy design might enhance note quality and usability while mitigating the unintended consequences of note bloat.

## Conclusions

This study found that outpatient progress notes grew significantly longer and more redundant across specialties between 2009 and 2018, providing a magnitude for the widely observed but poorly measured phenomena of note bloat. This study also found that the majority of note text was entered using templates and copy-paste rather than direct typing. It found an association between use of templates and copy-paste with longer and more redundant notes, and it also found that trainees and newer employees write longer notes. These findings suggest the factors driving note bloat are multifaceted and complex. Interventions aimed at halting note bloat may need to be similarly multifaceted. For example, simultaneously addressing note template design and clinician training. This study provided a baseline measure of note bloat in the decade following the passage of the landmark HITECH Act. It remains to be seen how recent policy changes affect clinical notes in the future.

## Notes

Supplement. **eTable 1.** Sample Sizes for Note Length, Note Redundancy, and Note Text Source Analyses by Specialty and Specialty Type **eTable 2.** Note Length, Note Redundancy, and Source of Note Text by Specialty and Specialty Type **eTable 3.** Parameter Estimates for Factors in Mixed-Effect Linear Models of Note Length and Note Redundancy Using Data From 2009-2018 and Only 2017-2018

## References

[1] Blumenthal D. Launching HITECH. N Engl J Med. 2010;362(5):382-385. doi:10.1056/NEJMp0912825 20042745

[2] Chaudhry B, Wang J, Wu S, . Systematic review: impact of health information technology on quality, efficiency, and costs of medical care. Ann Intern Med. 2006;144(10):742-752. doi:10.7326/0003-4819-144-10-200605160-00125 16702590

[3] Goldzweig CL, Towfigh A, Maglione M, Shekelle PG. Costs and benefits of health information technology: new trends from the literature. Health Aff (Millwood). 2009;28(2):w282-w293. doi:10.1377/hlthaff.28.2.w282 19174390

[4] Buntin MB, Burke MF, Hoaglin MC, Blumenthal D. The benefits of health information technology: a review of the recent literature shows predominantly positive results. Health Aff (Millwood). 2011;30(3):464-471. doi:10.1377/hlthaff.2011.0178 21383365

[5] Black AD, Car J, Pagliari C, . The impact of eHealth on the quality and safety of health care: a systematic overview. PLoS Med. 2011;8(1):e1000387. doi:10.1371/journal.pmed.1000387 21267058 PMC3022523

[6] Jones SS, Rudin RS, Perry T, Shekelle PG. Health information technology: an updated systematic review with a focus on meaningful use. Ann Intern Med. 2014;160(1):48-54. doi:10.7326/M13-1531 24573664

[7] Sinsky C, Colligan L, Li L, . Allocation of physician time in ambulatory practice: a time and motion study in 4 specialties. Ann Intern Med. 2016;165(11):753-760. doi:10.7326/M16-0961 27595430

[8] Arndt BG, Beasley JW, Watkinson MD, . Tethered to the EHR: primary care physician workload assessment using EHR event log data and time-motion observations. Ann Fam Med. 2017;15(5):419-426. doi:10.1370/afm.2121 28893811 PMC5593724

[9] Overhage JM, McCallie D Jr. Physician time spent using the electronic health record during outpatient encounters: a descriptive study. Ann Intern Med. 2020;172(3):169-174. doi:10.7326/M18-3684 31931523

[10] Hartzband P, Groopman J. Off the record—avoiding the pitfalls of going electronic. N Engl J Med. 2008;358(16):1656-1658. doi:10.1056/NEJMp0802221 18420497

[11] Hirschtick RE. A piece of my mind: copy-and-paste. JAMA. 2006;295(20):2335-2336. doi:10.1001/jama.295.20.2335 16720812

[12] Hirschtick RE. A piece of my mind: John Lennon’s elbow. JAMA. 2012;308(5):463-464. doi:10.1001/jama.2012.8331 22851112

[13] Gantzer HE, Block BL, Hobgood LC, Tufte J. Restoring the story and creating a valuable clinical note. Ann Intern Med. 2020;173(5):380-382. doi:10.7326/M20-0934 32658567

[14] Kuhn T, Basch P, Barr M, Yackel T; Medical Informatics Committee of the American College of Physicians. Clinical documentation in the 21st century: executive summary of a policy position paper from the American College of Physicians. Ann Intern Med. 2015;162(4):301-303. doi:10.7326/M14-2128 25581028

[15] Payne TH, Corley S, Cullen TA, . Report of the AMIA EHR-2020 Task Force on the status and future direction of EHRs. J Am Med Inform Assoc. 2015;22(5):1102-1110. doi:10.1093/jamia/ocv066 26024883 PMC5009932

[16] Shoolin J, Ozeran L, Hamann C, Bria W II. Association of Medical Directors of Information Systems consensus on inpatient electronic health record documentation. Appl Clin Inform. 2013;4(2):293-303. doi:10.4338/ACI-2013-02-R-0012 23874365 PMC3716423

[17] Goldstein IH, Hwang T, Gowrisankaran S, Bales R, Chiang MF, Hribar MR. Changes in electronic health record use time and documentation over the course of a decade. Ophthalmology. 2019;126(6):783-791. doi:10.1016/j.ophtha.2019.01.011 30664893 PMC6534421

[18] Downing NL, Bates DW, Longhurst CA. Physician burnout in the electronic health record era: are we ignoring the real cause? Ann Intern Med. 2018;169(1):50-51. doi:10.7326/M18-0139 29801050

[19] Siegler EL, Adelman R. Copy and paste: a remediable hazard of electronic health records. Am J Med. 2009;122(6):495-496. doi:10.1016/j.amjmed.2009.02.010 19486708

[20] Weis JM, Levy PC. Copy, paste, and cloned notes in electronic health records: prevalence, benefits, risks, and best practice recommendations. Chest. 2014;145(3):632-638. doi:10.1378/chest.13-0886 24590024

[21] O’Donnell HC, Kaushal R, Barrón Y, Callahan MA, Adelman RD, Siegler EL. Physicians’ attitudes towards copy and pasting in electronic note writing. J Gen Intern Med. 2009;24(1):63-68. doi:10.1007/s11606-008-0843-2 18998191 PMC2607489

[22] Centers for Medicare & Medicaid Services. Proposed policy, payment, and quality provisions changes to the Medicare physician fee schedule for calendar year 2019. Published July 12, 2018. Accessed November 6, 2019. https://www.cms.gov/newsroom/fact-sheets/proposed-policy-payment-and-quality-provisions-changes-medicare-physician-fee-schedule-calendar-year-3

[23] Centers for Medicare & Medicaid Services. Finalized policy, payment, and quality provisions changes to the Medicare physician fee schedule for calendar year 2020. Published November 1, 2019. Accessed July 21, 2020. https://www.cms.gov/newsroom/fact-sheets/finalized-policy-payment-and-quality-provisions-changes-medicare-physician-fee-schedule-calendar

[24] Tsou AY, Lehmann CU, Michel J, Solomon R, Possanza L, Gandhi T. Safe practices for copy and paste in the EHR. systematic review, recommendations, and novel model for health it collaboration. Appl Clin Inform. 2017;8(1):12-34. 28074211 10.4338/ACI-2016-09-R-0150 PMC5373750

[25] Lowry LZ, Ramaiah M, Prettyman SS, Examining the Copy and Paste Function in the Use of Electronic Health Records. National Institute of Standards and Technology; 2017. doi:10.6028/NIST.IR.8166

[26] World Medical Association. World Medical Association Declaration of Helsinki: ethical principles for medical research involving human subjects. JAMA. 2013;310(20):2191-2194. doi:10.1001/jama.2013.281053. 24141714

[27] Cohen AM, Chamberlin S, Deloughery T, . Detecting rare diseases in electronic health records using machine learning and knowledge engineering: case study of acute hepatic porphyria. PLoS One. 2020;15(7):e0235574. doi:10.1371/journal.pone.0235574 32614911 PMC7331997

[28] Wrenn JO, Stein DM, Bakken S, Stetson PD. Quantifying clinical narrative redundancy in an electronic health record. J Am Med Inform Assoc. 2010;17(1):49-53. doi:10.1197/jamia.M3390 20064801 PMC2995640

[29] Efron B, Tibshirani R. An Introduction to the Bootstrap. Chapman & Hall; 1998.

[30] Ren S, Lai H, Tong W, Aminzadeh M, Hou X, Lai S. Nonparametric bootstrapping for hierarchical data. J Appl Stat. 2010;37(9):1487-1498. doi:10.1080/02664760903046102

[31] Association of American Medical Colleges. Specialty profiles. Accessed July 21, 2020. https://www.aamc.org/cim/explore-options/specialty-profiles

[32] Hribar MR, Rule A, Huang AE, . Redundancy of progress notes for serial office visits. Ophthalmology. 2020;127(1):134-135. doi:10.1016/j.ophtha.2019.06.015 31358388 PMC6925342

[33] Zhang R, Pakhomov S, McInnes BT, Melton GB. Evaluating measures of redundancy in clinical texts. AMIA Annu Symp Proc. 2011;2011:1612-1620. 22195227 PMC3243221

[34] Singh H, Giardina TD, Meyer AND, Forjuoh SN, Reis MD, Thomas EJ. Types and origins of diagnostic errors in primary care settings. JAMA Intern Med. 2013;173(6):418-425. doi:10.1001/jamainternmed.2013.2777 23440149 PMC3690001

[35] Cohen R, Elhadad M, Elhadad N. Redundancy in electronic health record corpora: analysis, impact on text mining performance and mitigation strategies. BMC Bioinformatics. 2013;14(1):10. doi:10.1186/1471-2105-14-10 23323800 PMC3599108

[36] Wang MD, Khanna R, Najafi N. Characterizing the source of text in electronic health record progress notes. JAMA Intern Med. 2017;177(8):1212-1213. doi:10.1001/jamainternmed.2017.1548 28558106 PMC5818790

[37] Henriksen BS, Goldstein IH, Rule A, . Electronic health records in ophthalmology: source and method of documentation. Am J Ophthalmol. 2020;211:191-199. doi:10.1016/j.ajo.2019.11.030 31811860 PMC7073273

[38] Berdahl CT, Moran GJ, McBride O, Santini AM, Verzhbinsky IA, Schriger DL. Concordance between electronic clinical documentation and physicians’ observed behavior. JAMA Netw Open. 2019;2(9):e1911390. doi:10.1001/jamanetworkopen.2019.11390 31532513 PMC6751766

[39] Hendrickson MA, Melton GB, Pitt MB. The review of systems, the electronic health record, and billing. JAMA. 2019;322(2):115-116. doi:10.1001/jama.2019.5667 31173055

[40] Vawdrey DK. Assessing usage patterns of electronic clinical documentation templates. AMIA Annu Symp Proc. 2008;758-762. 18998863 PMC2656104

[41] Wilcox AB, Narus SP, Bowes WA III. Using natural language processing to analyze physician modifications to data entry templates. Proc AMIA Symp. 2002;899-903. 12463955 PMC2244350

[42] Rule A, Goldstein IH, Chiang MF, Hribar MR. Clinical documentation as end-user programming. Proc SIGCHI Conf Hum Factor Comput Syst. 2020;2020. Published online April 21, 2020. doi:10.1145/3313831.3376205 33629079 PMC7901830

[43] Aylor M, Campbell EM, Winter C, Phillipi CA. Resident notes in an electronic health record: a mixed-methods study using a standardized intervention with qualitative analysis. Clin Pediatr (Phila). 2017;56(3):257-262. doi:10.1177/0009922816658651 27400934

[44] Kahn D, Stewart E, Duncan M, . A prescription for note bloat: an effective progress note template. J Hosp Med. 2018;13(6):378-382. doi:10.12788/jhm.2898 29350222

[45] Dean SM, Eickhoff JC, Bakel LA. The effectiveness of a bundled intervention to improve resident progress notes in an electronic health record. J Hosp Med. 2015;10(2):104-107. doi:10.1002/jhm.2283 25425386 PMC4498456

