# Supplementary material for: Length and Redundancy of Outpatient Progress Notes Across a Decade at an Academic Medical Center
Source: JAMA Netw Open. 2021 Jul 19;4(7):e2115334. doi: 10.1001/jamanetworkopen.2021.15334 (PMC8290305; doi:10.1001/jamanetworkopen.2021.15334)
Supplement: Supplement. — eTable 1. Sample Sizes for Note Length, Note Redundancy, and Note Text Source Analyses by Specialty and Specialty Type eTable 2. Note Length, Note Redundancy, and Source of Note Text by Specialty and Specialty Type eTable 3. Parameter Estimates for Factors in Mixed-Effect Linear Models of Note Length and Note Redundancy Using Data From 2009-2018 and Only 2017-2018 [file jamanetwopen-e2115334-s001.pdf]

Supplemental Online Content

Rule A, Bedrick S, Chiang MF, Hribar MR. Length and redundancy of outpatient progress notes across a decade at an academic medical center. *JAMA Netw Open*. 2021;4(7):e2115334. doi:10.1001/jamanetworkopen.2021.15334

eTable 1. Sample Sizes for Note Length, Note Redundancy, and Note Text Source Analyses by Specialty and Specialty Type
eTable 2. Note Length, Note Redundancy, and Source of Note Text by Specialty and Specialty Type
eTable 3. Parameter Estimates for Factors in Mixed-Effect Linear Models of Note Length and Note Redundancy Using Data From 2009-2018 and Only 2017-2018

This supplemental material has been provided by the authors to give readers additional information about their work.

© 2021 Rule A et al. *JAMA Network Open.*

eTable1. Sample Sizes for Note Length, Note Redundancy, and Note Text Source Analyses by Specialty and Specialty Type

Specialty		Note Length Analysis			Note Redundancy Analysis			Note Text Source Analysis		
**Specialty Name**	**Specialty Type**	**# Notes**	**# Auth.**	**# Patients**	**# Note Pairs**	**# Auth.**	**# Patients**	**# Notes**	**# Auth.**	**# Patients**
Family Practice	Primary Care	706268	1192	93906	272934	396	51992	191466	552	50117
Internal Medicine	Primary Care	292212	759	31319	135131	378	20767	66258	290	16440
Pediatrics	Primary Care	238879	793	28410	59314	318	15717	53704	320	12053
Ophthalmology	Adult	232601	349	36567	97618	242	20117	54132	169	16883
Obstetrics & Gynecology	Primary Care	193320	689	25478	59368	278	12744	40435	239	9901
Orthopedics	Surgical	140035	317	28677	55388	93	16332	31091	132	10003
Dermatology	Adult	138428	427	36324	49405	140	13642	32747	140	14803
Hematology & Oncology	Adult	102665	401	10077	53777	157	6296	22827	158	4333
Otolaryngology	Adult	64138	278	20358	26081	115	8376	14313	95	6963
Cardiology	Adult	60460	265	12875	22961	135	6490	17373	126	5906
Endocrinology	Adult	60282	382	10176	29274	98	5825	12539	122	4164
Neurology	Adult	42504	238	10829	21732	168	5273	9561	115	3995
Pain Management	Adult	41440	157	8845	14174	47	2475	12765	59	2510
Surgery	Surgical	33046	566	9203	12307	152	4251	6895	187	2817
Urology	Surgical	28674	151	8577	11991	49	3807	6635	30	2879
Rheumatology	Adult	26087	232	5759	15633	57	3076	6323	74	2258
Plastic Surgery	Surgical	23854	158	6123	7174	56	2286	6253	45	1936
Pediatric Hem. & Onc.	Pediatric	23663	169	1427	9559	63	824	5435	58	604
Neurological Surgery	Surgical	17482	139	5350	4899	77	2010	3987	64	1872
Gastroenterology	Adult	16320	99	7365	5967	56	2349	4005	50	2566
Vascular Surgery	Surgical	15613	318	3642	1867	81	996	3116	105	1322
Radiation Oncology	Adult	15393	72	2847	6434	49	1608	3756	35	913
Surgical Oncology	Surgical	14288	307	3411	6475	65	1811	3105	76	1301
Pulmonary Disease	Adult	14054	183	4405	6422	59	1907	2133	67	1069
Nephrology	Adult	13361	113	2941	8042	63	1661	3098	48	1292
Hepatobiliary Surgery	Surgical	11879	260	1743	4288	59	920	3132	75	931
Hepatology	Adult	11178	86	3295	6601	16	1727	1972	35	1174
Sleep Medicine	Adult	11032	45	5461	1128	14	606	58	10	53
Pediatric Gastro.	Pediatric	10130	115	3100	5960	19	1590	2582	41	1098
Pediatric Cardiology	Pediatric	10021	47	3235	4852	28	1188	2233	26	1228
Infectious Disease	Adult	9222	114	2992	4566	47	1418	1752	56	885
Pediatric Orthopedics	Surgical	8454	135	3772	1830	35	1082	2029	53	1014
Pediatric Nephrology	Pediatric	8102	78	1086	4806	10	756	2247	25	528
Pediatric Neurology	Pediatric	8026	209	2274	3533	29	1021	1782	35	851

© 2021 Rule A et al. *JAMA Network Open.*

Pediatric Pulmonology	Pediatric	7839	93	1415	3045	8	805	1767	25	611
Facial Plastic Surgery	Surgical	7162	40	1899	3576	22	1011	1719	9	690
Pediatric Surgery	Surgical	7017	132	2522	1882	29	777	1454	24	733
Pediatric Urology	Surgical	6295	53	2307	2363	22	947	1681	26	876
Pediatric Endocrinology	Pediatric	6112	194	1878	2783	23	824	1468	67	747
Heart Transplant	Surgical	5918	63	816	2130	21	523	1156	10	231
Pediatric Neuro. Surgery	Surgical	5523	44	1504	779	33	450	1471	23	571
Oral & Max. Surgery	Surgical	4665	53	2063	1355	35	561	11	5	11
Allergy & Immunology	Adult	3825	147	2224	1310	5	481	723	24	566
Cardiac Surgery	Surgical	2954	48	1500	547	18	381	728	18	385
Thoracic Surgery	Surgical	2946	111	1016	311	21	211	695	31	305
Pediatric Inf. Dis.	Pediatric	1433	58	441	873	6	178	305	15	120
	Primary Care	1430679	2945	150880	526747	1333	91992	351863	1319	79915
	Adult	891664	2863	95347	383116	1483	54401	206712	1237	45546
	Surgical	306516	1739	54909	108386	690	29418	68261	681	21038
	Pediatric	75941	745	13231	34196	210	6298	18081	294	5110
	All	2704800	6228	182487	1052445	3460	123734	644917	3049	100950

Auth. = Authors; Hem. & Onc. = Hematology & Oncology; Gastro. = Gastroenterology; Neuro. = Neurological; Max = Maxillofacial; Inf. Dis. = Infectious Diseases

© 2021 Rule A et al. *JAMA Network Open.*

eTable 2. Note Length, Note Redundancy, and Source of Note Text by Specialty and Specialty Type

Specialty	Note Length			Note Redundancy			Note Text Source			
	2009 Median (Words)	2018 Median (Words)	% Increase 2009-2018	2009 Median (%)	2018 Median (%)	%-point Increase 2009-2018	2018 Typed (%Chars)	2018 Copied (%Chars)	2018 Template (%Chars)	2018 Non-Provider (%Chars)
Allergy & Immunology	272	348	28.2	68.0	80.8	12.8	25.0	4.0	70.9	1.8
Cardiac Surgery	670	833	24.3	49.9	54.4	4.5	23.9	18.7	57.4	24.5
Cardiology	659	1211	83.7	61.9	72.8	10.9	22.7	37.2	40.1	17.3
Dermatology	270	548	103.0	54.5	66.2	11.7	29.5	22.5	48.0	56.1
Endocrinology	619	900	45.4	53.0	65.5	12.5	26.4	24.1	49.6	4.4
Facial Plastic Surgery[b]	181	197	8.8	42.7	44.8	2.1	41.1	12.5	46.4	59.1
Family Practice	345	506	46.7	39.2	47.8	8.6	33.2	5.8	61.0	7.7
Gastroenterology	584	1289	120.6	40.6	59.4	18.8	29.0	22.3	48.8	23.2
Heart Transplant	565	1604	183.9	64.6	74.9	10.4	18.0	53.6	28.4	3.6
Hematology & Oncology	344	1175	241.6	76.3	77.7	1.4	16.5	46.5	37.0	6.8
Hepatobiliary Surgery	787	987	25.4	65.2	77.9	12.7	20.3	17.8	61.8	3.4
Hepatology	705	1008	43.0	67.8	70.0	2.2	22.4	26.0	51.6	0.0
Infectious Disease	1063	1080	1.6	61.0	54.6	-6.4	34.6	18.2	47.2	0.1
Internal Medicine	695	964	38.7	49.8	54.2	4.4	33.9	6.1	59.9	11.8
Nephrology	674	1013	50.2	56.1	67.4	11.3	23.7	19.6	56.7	15.3
Neurological Surgery	284	830	192.1	26.7	61.2	34.5	32.6	22.2	45.2	14.6
Neurology	743	1275	71.6	50.5	70.4	19.8	26.4	35.4	38.2	9.8
Obstetrics & Gynecology	251	676	169.9	44.7	54.7	9.9	32.8	18.0	49.2	27.0
Ophthalmology	344	583	69.5	54.6	74.7	20.1	11.4	13.7	74.9	56.1
Oral & Max. Surgery[a]	196	264	34.7	18.2	53.3	35.1	30.9	7.8	61.3	16.0
Orthopedics	292	393	34.8	33.8	42.6	8.8	30.4	19.1	50.5	21.7
Otolaryngology	335	694	107.2	45.7	62.2	16.5	24.7	16.0	59.3	22.0
Pain Management	345	467	35.4	58.4	64.9	6.5	33.3	26.8	39.9	16.0
Pediatric Cardiology	540	835	54.6	58.6	70.0	11.4	24.0	38.7	37.2	9.2
Pediatric Endocrinology	711	973	36.8	58.1	68.3	10.1	27.8	42.9	29.3	7.8
Pediatric Gastro.	680	1044	53.5	49.4	64.1	14.7	18.8	18.4	62.8	9.4

© 2021 Rule A et al. *JAMA Network Open.*

Pediatric Hem. - Onc.	706	1180	67.1	75.5	78.3	2.8	15.3	58.7	26.0	3.9
Pediatric Inf. Dis. [b]	548	1111	102.7	62.5	62.5	0.0	36.2	19.1	44.7	1.2
Pediatric Nephrology	773	1315	70.1	45.3	59.2	13.9	23.1	19.2	57.8	7.8
Pediatric Neuro. Surgery	299	460	54.1	26.1	38.1	12.0	36.0	16.1	47.8	7.1
Pediatric Neurology	795	1199	50.8	41.5	63.6	22.1	24.2	35.4	40.4	11.2
Pediatric Orthopedics	372	357	-4.2	29.2	58.4	29.2	41.1	16.4	42.5	42.8
Pediatric Pulmonology	688	1109	61.3	65.3	56.8	-8.5	21.5	15.9	62.6	6.5
Pediatric Surgery	249	692	178.5	27.2	41.6	14.4	29.9	14.9	55.2	54.5
Pediatric Urology[b]	481	684	42.2	31.0	46.1	15.1	29.4	14.2	56.4	14.1
Pediatrics	348	590	69.4	37.5	46.8	9.3	37.0	8.4	54.7	15.6
Plastic Surgery	167	280	67.7	25.2	65.1	39.9	37.3	18.9	43.9	19.7
Pulmonary Disease	1029	1365	32.7	34.1	66.0	31.9	28.8	14.5	56.7	3.1
Radiation Oncology	381	450	18.1	58.0	58.9	0.9	17.1	19.5	63.4	17.2
Rheumatology	775	1233	59.1	59.1	70.2	11.1	23.3	27.3	49.4	5.1
Sleep Medicine	446	1252	180.7	52.6	65.0	12.4	28.3	14.3	57.4	5.9
Surgery	280	710	153.4	38.7	52.2	13.5	25.9	14.9	59.1	33.2
Surgical Oncology	724	1166	61.0	41.8	66.7	24.9	14.9	27.9	57.2	11.4
Thoracic Surgery	764	765	0.1	47.1	57.0	9.8	27.6	14.7	57.7	2.3
Urology	464	566	22.1	54.4	66.4	11.9	21.6	27.2	51.1	3.9
Vascular Surgery	328	573	74.7	38.2	42.2	4.0	37.9	19.4	42.7	8.0
Adult	421	775	84.1	57.5	70.3	12.8	21.7	24.6	53.7	30.2
Pediatric	642	1013	57.8	60.0	67.2	7.2	23.1	36.3	40.6	11.2
Primary	394	589	49.5	43.5	50.1	6.5	33.8	7.5	58.7	11.5
Surgical	330	555	68.2	35.8	52.5	16.6	29.8	19.1	51.1	20.6
All	401	642	60.1	47.9	58.8	10.9	29.4	14.7	55.9	18.2

[a]2017 data used in place of 2018 data
[b]2010 data used in place of 2009 data
Hem. & Onc. = Hematology & Oncology; Gastro. = Gastroenterology; Neuro. = Neurological; Max = Maxillofacial; Inf. Dis. = Infectious Diseases

© 2021 Rule A et al. *JAMA Network Open.*

eTable 3. Parameter Estimates for Factors in Mixed-Effect Linear Models of Note Length and Note Redundancy Using Data From 2009-2018 and Only 2017-2018

	Note Length (% increase)		Note Redundancy (%-point increase)	
	**2009 - 2018 Data**	**2017-2018 Data**	**2009 - 2018 Data**	**2017-2018 Data**
**Factor**	**Est. (95% CI)**	**Est. (95% CI)**	**Est. (95% CI)**	**Est. (95% CI)**
Encounter Year (per year)	2.4 (1.4 to 3.5)	0.5 (-1.0 to 2.0)	0.7 (0.5 to 1.0)	0.3 (-0.03 to 0.7)
Author Start Year (per year)	1.8 (1.3 to 2.4)	2.0 (1.3 to 2.6)	0.2 (0.03 to 0.3)	0.4 (0.3 to 0.5)
Author is Trainee	26.3 (25.8 to 26.7)	5.0 (4.1 to 5.9)	-7.0 (-6.7 to -7.3)	-4.0 (-3.5 to -4.6)
Billing: Level 4 vs. Level 3	27.9 (27.7 to 28.2)	26.1 (25.7 to 26.5)	-1.9 (-1.8 to -2.0)	-2.6 (-2.4 to -2.8)
New Visit vs. Return Visit	32.2 (31.9 to 32.4)	29.3 (28.9 to 29.8)	-16.7 (-16.2 to -17.1)	-15.6 (-14.8 to -16.2)
Number of Prior Visits (per visit)	0.1 (0.1 to 0.1)	0.1 (0.1 to 0.1)	0.04 (0.04 to 0.04)	0.02 (0.02 to 0.02)
% Note Text by non-Auth. (per 1%)[a]	-	0.5 (0.5 to 0.5)	-	-0.03 (-0.03 to -0.04)
% Note Text Copied (per 1%)[a]	-	1.5 (1.5 to 1.5)	-	0.6 (0.6 to 0.6)
% Note Text Templated (per 1%)[a]	-	1.6 (1.6 to 1.6)	-	0.4 (0.4 to 0.4)

[a] Note source data were only available for the years of 2017-2018, so not included as factors in the models using 2009-2018 data.
Est. = Estimate; non-Auth. = non primary author

© 2021 Rule A et al. *JAMA Network Open.*
